# Roles of *COMT*, *NPY* and *GCH1* in acute and chronic pain/stress response

**DOI:** 10.1186/1744-8069-10-S1-O5

**Published:** 2014-12-15

**Authors:** David Goldman

**Affiliations:** 1 Laboratory of Neurogenetics, NIAAA, Rockville, MD 20852, USA

## 

Pain is a pervasive stressor often experienced chronically, and strongly modulated by the brain’s emotional circuitry. In recent years functional polymorphisms at several genes have been linked to pain response, encouraging the idea that both pain response and other aspects of emotionality can be better understood by genetic studies of acute and chronic pain, as illustrated by studies performed with several genes, two of which also alter anxiety and emotional responses. The functional *COMT* Val158Met locus, which had been tied to frontal cognitive function, trait anxiety and brain metabolic responses to emotional stimuli, was linked to the ability of acute pain to release endomorphin and displace ^11^C carfentanil binding following a pain challenge [1]. The anxiety-associated Met158 allele predicts both lower pain threshold and stronger affective response to pain. This finding was replicated in a large sample of women prospectively followed or temporomandibular joint pain and measured for experimental pain (Diatchenko et al). These investigators later showed that the *COMT* haplotype linkage is better understood via the epistatic interaction of alleles to alter translatability of *COMT* mRNA. GTP cyclohydrolase (*GCH1*) represents a second gene influencing pain (Tegeder et al, Nat Neuroscience). The gene was identified as a candidate via array expression in the rat neurotomy model. In humans, a functional haplotype predicting *GCH1* mRNA expression in lymphoblastoid cell lines. Consistent with the rat data, the high expression diplotype was linked to both clinical post lumbar surgery leg pain and to experimental pain in a large population of controls. Continuing the theme that genes that alter emotion can also alter pain responses, a functional polymorphism in the *NPY* (neuropeptide Y) propmoter alters both amygdala and hippocampal emotional responses as well as predicting ^11^C carfentanil displacement after a pain challenge [2].
